# Pharmacokinetic Evaluation of a Cinnamon Product on CYP2A6 Substrate Drugs: Application of a Novel Tool Involving the Nicotine Metabolite Ratio

**DOI:** 10.1002/cpt.70218

**Published:** 2026-02-01

**Authors:** Aiden‐Hung P. Nguyen, Deena L. Hadi, Daniel A. Todd, Preston K. Manwill, John R. White, Matthew E. Layton, Nadja B. Cech, Kenneth E. Thummel, Mary F. Paine

**Affiliations:** ^1^ College of Pharmacy and Pharmaceutical Sciences Washington State University Spokane WA USA; ^2^ Center of Excellence for Natural Product Drug Interaction Research Spokane WA USA; ^3^ Department of Chemistry and Biochemistry University of North Carolina at Greensboro Greensboro North Carolina USA; ^4^ Elson S. Floyd College of Medicine Washington State University Spokane WA USA; ^5^ Department of Pharmaceutics, School of Pharmacy University of Washington Seattle WA USA

## Abstract

Cinnamon (*Cinnamomum* spp.) is used as a culinary spice and dietary supplement. A major constituent, cinnamaldehyde, was previously shown to inactivate cytochrome P450 (CYP) 2A6 in vitro. A mechanistic static model predicted an ~5‐fold increase in the AUC of the CYP2A6 substrates nicotine and letrozole. Accordingly, the effects of a well‐characterized cinnamon (*Cinnamomum verum*) product on the pharmacokinetics of nicotine and letrozole were evaluated in 16 healthy, non‐nicotine using adults. They were administered a single dose of nicotine gum (2 mg) or letrozole tablet (2.5 mg) (baseline). After a sufficient washout (2–14 days), they self‐administered *C. verum* (2 g thrice daily) for 5 consecutive days. On Day 6, they were administered *C. verum* with nicotine or letrozole, followed by two more doses of *C. verum* (cinnamon exposure). Plasma was collected from 0 to 12 (nicotine) or 0–240 (letrozole) hours. The geometric mean plasma concentration vs. time profile for both drugs was nearly superimposable in the presence vs. absence of *C. verum*. The geometric mean ratio (GMR) [90% confidence interval] of the AUC of nicotine and letrozole in the presence to absence of cinnamon was 0.98 [0.96–1.12] and 1.11 [0.98–1.24], respectively (*P* > 0.16), indicating no interactions. Application of the “slope approach” involving the 3‐hydroxycotinine‐to‐cotinine ratio provided potential new mechanistic insight into CYP2A6 inhibition. The general lack of effect of a typical dosage of *C. verum* on the pharmacokinetics of nicotine and letrozole suggests that *C. verum* may be safe to consume with both drugs, as well as other CYP2A6 substrates.

**Study Highlights**

**WHAT IS THE CURRENT KNOWLEDGE ON THE TOPIC?**

*Cinnamomum verum* (*C. verum*) is a botanical dietary supplement that is purported to have multiple beneficial health effects. Cinnamaldehyde, a major constituent in *C. verum* and a time‐dependent inhibitor of cytochrome P450 2A6 (CYP2A6) in vitro, was predicted to increase the area under the plasma concentration vs. time curve of the clinically relevant CYP2A6 substrates nicotine and letrozole by 4‐ to 5‐fold.

**WHAT QUESTION DID THIS STUDY ADDRESS?**

What are the effects of a well‐characterized *C. verum* product on the clinical pharmacokinetics of nicotine and letrozole? Is the nicotine metabolite ratio at a single time point a robust tool to assess CYP2A6‐mediated drug interactions?

**WHAT DOES THIS STUDY ADD TO OUR KNOWLEDGE?**

The *C. verum* product had negligible effects on the pharmacokinetics of nicotine and letrozole in healthy adults. A novel analysis tool termed the slope approach was developed that provided additional mechanistic insight into CYP2A6 inhibition.

**HOW MIGHT THIS CHANGE CLINICAL PHARMACOLOGY OR TRANSLATIONAL SCIENCE?**

Results indicate that the *C. verum* product taken at a recommended dosage may be safe to consume with CYP2A6 substrates, filling a knowledge gap in the understudied area of natural product‐drug interactions. The slope approach may be applicable to other relevant enzyme‐mediated clinical pharmacokinetic drug interaction studies.

Sales of herbal supplements exceeded $12 billion in 2023 and are projected to increase by 10% annually.^1^ These and other natural products, derived from herbs, bacteria, or fungi, continue to grow in popularity due to their perceived “naturalness” and low toxicity.^2^ Despite increasing use, potential pharmacokinetic interactions between natural products and pharmaceutical medications remain understudied. The relative lack of well‐designed, controlled natural product‐drug interaction studies highlights potential patient safety concerns, including subtherapeutic drug effects, toxicities, and altered drug pharmacokinetic and pharmacodynamic properties.

Cinnamon (*Cinnamomum* spp.) is used worldwide as both a culinary spice and botanical dietary supplement, the latter of which ranked among the 20 top‐selling herbal supplements in the United States in 2023, with sales increasing by ~60% compared to 2022.^1^ Cinnamon is added to various products, ranging from foods (*e.g*., breakfast cereals and baked goods) to fragrances and essential oils, to improve taste or aroma. As a dietary supplement, cinnamon is commonly used for multiple purported health benefits, including antimicrobial, anti‐inflammatory, and blood glucose‐lowering effects.^3^, ^4^ Cinnamon contains numerous phytochemicals, including the phenylpropanoids cinnamaldehyde and methoxycinnamaldehyde, from which the flavor and scent of cinnamon emanate.

Phenylpropanoids constitute a diverse family of plant‐derived phytochemicals that have demonstrated various biological activities using in vitro and animal models, including those mentioned above.^5^, ^6^ Both cinnamaldehyde and methoxycinnamaldehyde were shown to be mechanism‐based inhibitors of human cytochrome P450 (CYP) 2A6 through heme degradation and apoprotein modification.^7^, ^8^ These low molecular weight compounds are relatively small and, based on the crystal structure of CYP2A6, enable them to fit in the small active site of the enzyme.^9^ Unlike competitive inhibitors, of which inhibition ceases upon their removal, mechanism‐based inhibitors inactivate the enzyme permanently, leading to protracted recovery of the enzyme and longer lasting effects, even after the inhibitor is removed.^10^, ^11^ Consequently, drug interactions with mechanism‐based inhibitors can last for several days. A clinically relevant natural product example is grapefruit juice, which contains furanocoumarins that are mechanism‐based inhibitors of intestinal CYP3A4, leading to numerous potential adverse drug interactions.^12^, ^13^ Relative to CYP3A4, the list of clinically relevant CYP2A6 substrates is very short. Two critical substrates include the established CYP2A6 probe, nicotine, and the anticancer agent letrozole. Using an in vitro‐to‐in vivo extrapolation approach, cinnamaldehyde and methoxycinnamaldehyde were predicted to increase the area under the plasma concentration vs. time curve (AUC) of both substrates by 4‐ to 5‐fold,^8^ well exceeding the FDA‐recommended cutoff for further in vivo investigation (1.25).^14^

Cinnamon comprises hundreds of species that range in abundance of various phytoconstituents, including cinnamaldehyde and methoxycinnamaldehyde, as well as the hepatotoxicant coumarin. *Cinnamomum verum* (*C. verum*) and *C. cassia* contain high cinnamaldehyde content and are the leading commercially available species.^15^ However, the German Federal Institute of Risk Management concluded that *cassia*‐type cinnamon products may pose a potential health risk due to coumarin potentially exceeding the total daily intake (0.1 mg/kg).^16^ Accordingly, most commercially available cinnamon supplements are prepared from *C. verum*. Based on the collective observations, the objective of the current work was to evaluate a well‐characterized, commercially available *C. verum* product as a precipitant of pharmacokinetic interactions with nicotine and letrozole in healthy adults. Results inform the safety of co‐consuming *C. verum* with CYP2A6 substrates and potential new mechanistic insight into CYP2A6 inhibition.

## METHODS

### Materials and chemicals

A *C. verum* product was obtained from the Ceylon Cinnamon Shop (Issaquah, WA). Cinnamaldehyde, methoxycinnamaldehyde, and coumarin were characterized using a Waters Acquity Ultra‐Performance Liquid Chromatography (UPLC) system (Waters, Milford, MA, USA) coupled to a Q Exactive Plus mass spectrometer (Thermo Fisher Scientific, Waltham, MA). Methods used to characterize the study material are detailed in the **Supplemental Material** **S1**. The product was in the form of capsules containing cinnamon bark powder. Each capsule contained 4.135 ± 0.450 mg of cinnamaldehyde, 0.275 ± 0.027 mg of methoxycinnamaldehyde, and 0.0130 ± 0.0007 mg of coumarin (**Table** **S1**). The product underwent contamination testing for heavy metals, microbial and fungal contaminants, pesticides, and volatile organic compounds. All substances measured were within allowable limits (**Supplemental Material** **S1**).

### Clinical pharmacokinetic study

#### Participants

The clinical study protocol and informed consent form were approved by the Washington State University (WSU) Institutional Review Board. The study took place at the Human Research Clinic (HRC) on the WSU Health Sciences Campus under Investigational New Drug status (#157412) and in accordance with the Code of Federal Regulations on the Protection of Human Subjects (45 CFR Part 46). The study was registered on the ClinicalTrials.gov database (NCT05157672). This crossover, open‐label study involved eight males and eight non‐pregnant, non‐lactating females aged 18–64 who were non‐nicotine users and in good health. Interested individuals provided written informed consent and Health Insurance Portability and Accountability Act information before screening. Eligibility was determined during the screening visit, which included a physical examination, disclosure of medical history and medication use, collection and laboratory testing of blood and urine samples, a urine pregnancy test for females, and review of inclusion/exclusion criteria (**Table** **S2**).

#### Study design

A detailed timeline of the study procedures is provided (**Figure** **S2**). Briefly, following an overnight fast, participants were administered a single dose (2 g) of the *C. verum* product. After at least 7 days, following an overnight fast, participants were administered a single dose (2 mg) of nicotine gum (Rugby, La Vergne, TN) or a single tablet (2.5 mg) of letrozole (Breckenridge, Berkeley Heights, NJ) with 240 mL water. They were instructed to chew the gum for 20 seconds, then position the gum between the inner cheek and gumline for 40 seconds. They repeated the process for 30 minutes. Following a washout period of at least 24 hours (nicotine) or 14 days (letrozole), participants self‐administered *C. verum* (2 g) three times daily, approximately every 4 hours, for five consecutive days, monitored via a secure video link. On Day 6, participants returned to the HRC, where they were administered *C. verum* (2 g) 30 minutes prior to a single dose of nicotine gum or letrozole; they were administered *C. verum* two additional times during the study day.

Blood (~5 mL) was drawn into BD K_3_ EDTA‐containing vacutainer tubes (Fisher Scientific, Pittsburgh, PA) from an indwelling venous catheter (placed in an arm vein) 30 minutes before and 0.5, 1, 1.5, 2, 3, 4, 6, 8, 10, and 12 hours after nicotine or letrozole was administered (**Figure** **S2**). Participants fasted until after the 4‐hour blood collection, when they were provided lunch. During Arms 1, 3, and 5, they returned to the HRC at up to 240 hours for blood collection *via* venipuncture. The tubes were immediately placed on ice after each collection for 30 minutes, then centrifuged at 1,600 ***g*** for 10 minutes to separate plasma from red blood cells. Plasma samples were stored at −80°C until bioanalysis.

### Bioanalysis of plasma for nicotine, nicotine metabolites, and letrozole

#### Nicotine and nicotine metabolites

Plasma samples were analyzed for nicotine (Thermo Fisher Scientific, Waltham, MA), cotinine, and 3‐hydroxycotinine (Toronto Research Chemicals, Vaughan, ON) using a previously published LC–MS/MS method.^17^ Briefly, 600 μL of methanol containing 0.1% acetic acid and 250 ng/mL of each internal standard (*d*_*4*_‐nicotine, *d*_*3*_‐cotinine, and *d*_*3*_‐3‐hydroxycotinine; Cerilliant, Round Rock, TX) were added to 200 μL of plasma. Samples were vortexed and centrifuged at 14,000 ***g*** for 10 min at 4°C. The supernatant was mixed with 50 μL of 1% HCl in methanol and evaporated under nitrogen gas at 37°C. The dried residue was dissolved in 100 μL of mobile phase, vortexed, and centrifuged at 14,000 ***g*** for 10 min at 4°C. Supernatant (3 μL) was injected into the LC–MS/MS. Calibration curves ranging from 1.2 to 350 ng/mL for each analyte were prepared fresh for each run. Inter‐day variability was determined using quality controls at low, medium, and high concentrations over a 6‐month period; all analytes varied by <10%. Additional details are provided (**Table** **S3**).

#### Letrozole

Plasma samples were analyzed for letrozole (Sigma Aldrich, St. Louis, MO) using a UPLC‐MS/MS system consisting of a Shimadzu (Columbia, MD) ultrahigh‐performance liquid chromatograph (UPLC) coupled with a triple quadrupole AB‐SciEx 6500 series mass spectrometer (Framingham, MA). Briefly, 200 μL of ice‐cold acetonitrile containing the internal standard, *d*_*4*_‐letrozole‐d_4_ (60 nM) (Cayman Chemical, Ann Arbor, MI), was added to 100 μL of plasma. Samples were vortexed and centrifuged at 2300 ***g*** for 15 min at 4°C. Supernatant (10 μL) was injected into the UPLC‐MS/MS for analysis. Calibration curves ranging from 1.56 to 200 nM were prepared fresh for each run. The average inter‐day variability for each concentration in the calibration curve ranged from 2.6–7.3%. Additional details are provided (**Table** **S3**).

### Pharmacokinetic analysis

The pharmacokinetics of nicotine and letrozole were obtained *via* non‐compartmental analysis methods using Phoenix WinNonlin™ (version 8.4; Certara, Radnor, PA) as described.^18^, ^19^ The following were recovered: maximum plasma concentration (*C*_max_), time to reach *C*_max_ (*t*_max_), area under the plasma concentration *vs*. time curve (AUC) from time 0 to the last measured concentration (AUC_0−t_), terminal half‐life (t_1/2_), and apparent clearance (CL/F). CYP2A6‐dependent metabolic activity was assessed using the nicotine metabolite ratio (NMR), calculated as the ratio of the concentration of 3‐hydroxycotinine to that of cotinine at 4, 6, and 8 hours. Partial AUC from 3 to 8 hours (AUC_3−8h_) for each metabolite was determined to capture the elimination phase of cotinine and the formation phase of 3‐hydroxycotinine.^20^ The inhibitory effects of *C. verum* on nicotine metabolism were further evaluated using the novel “slope approach”, which entailed determining the slope of the NMR vs. time plot using (1) all time points and (2) the time interval that was most linear (i.e., lacked curvature, beginning at the *t*_max_ of cotinine and ending at the *t*_max_ of 3‐hydroxycotinine). Analytes below the limit of quantification were treated as missing. Due to insufficient data points at later times for the cotinine and 3‐hydroxycotinine profiles, recovery of robust pharmacokinetic endpoints was not possible for these metabolites. Pharmacokinetic data are reported as geometric means (90% confidence interval) or median (range) as appropriate.

### Statistical analysis

Sample size was determined using a two‐tailed paired t‐test analysis for comparing the primary endpoint (geometric mean ratio (GMR) of nicotine or letrozole AUC in the presence to absence of *C. verum*). Assuming 26% intra‐individual variability (coefficient of variation percentage (CV%)) in nicotine AUC,^21^ 16 evaluable participants were required to detect a 20% change in AUC with a Type I error of 0.05 and 80% power. Assuming 16% intra‐individual variability (CV%) in letrozole AUC,^22^ 10 evaluable participants were required to detect a 20% change in AUC with a Type I error of 0.05 and >90% power. The higher power for letrozole was selected to ensure a higher degree of confidence in detecting an interaction due to letrozole being an anti‐cancer medication. The pre‐defined no‐effect range for both interactions was 0.80–1.25. Specifically, if the GMR (with CI) lay within this range, then a pharmacokinetic interaction was not evident. True differences in GMRs were assessed using the paired Student's t‐test, with *P*‐values <0.05 considered significant.

## RESULTS

### Participants, safety, and tolerability

Of the 16 participants enrolled in the study, 6, 16, 14, 16, and 10 completed Arms 1, 2, 3, 4, and 5, respectively. One male was denied continuing the study due to a false report of nicotine use and was replaced with another eligible participant. A total of six participants were excluded from the nicotine pharmacokinetic analysis: three used nicotine within the 24‐hour period before nicotine administration (based on cotinine being detected in their plasma during Arm 2), two exhibited nicotine plasma concentrations below the limit of quantification (LOQ) after 2 hours of nicotine administration, and one chewed the gum for 1 hour during Arm 2. Participants who completed the study self‐identified as White or Caucasian, Asian, Black or African American, and Native American. Most of them were White or Caucasian. The median (range) age was 34 (22–57) years. Both object drugs and the *C. verum* product were well tolerated. No participant experienced a serious adverse event. Four participants reported an adverse event during Arms 3 and 5 (headache, lightheadedness, hot flashes, and nausea) that did not result in study discontinuation.

### Pharmacokinetics of nicotine and letrozole

**Figure 1:**
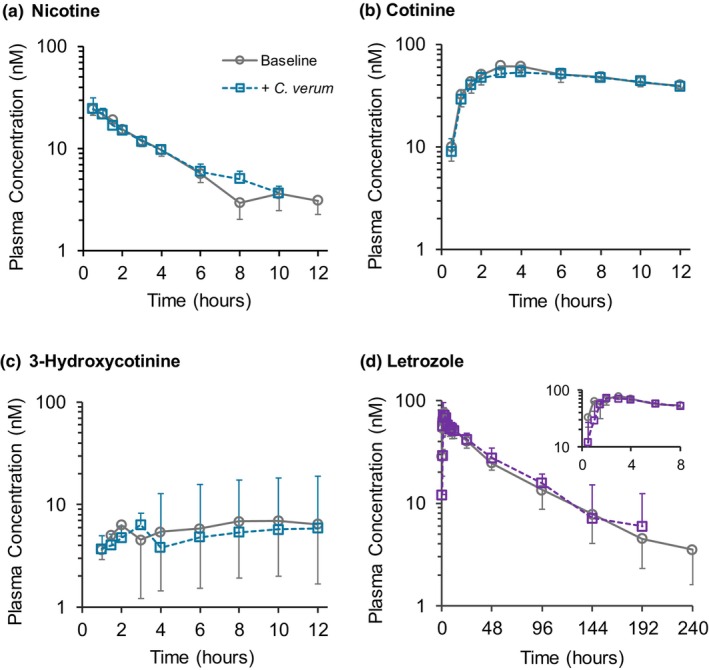
Geometric mean plasma concentration vs. time profiles for (**a**) nicotine (*n* = 10), (**b**) cotinine (*n* = 11), (**c**) 3‐hydroxycotinine (*n* = 11), and (**d**) letrozole (*n* = 10) following administration of a single dose of nicotine gum (2 mg) or letrozole tablet (2.5 mg) alone (circles) and after exposure to a well‐characterized *C. verum* product (squares) (2 g thrice daily for 6 days). Error bars denote 90% confidence intervals. The inset in the letrozole panel shows the 0–8 hour time period. The missing data point for nicotine at 12 hours (**a**) and letrozole at 240 hours (**d**), both in the presence of C. verum, represent >50% of the participants with plasma concentrations below the limit of quantification.

**Figure 2:**
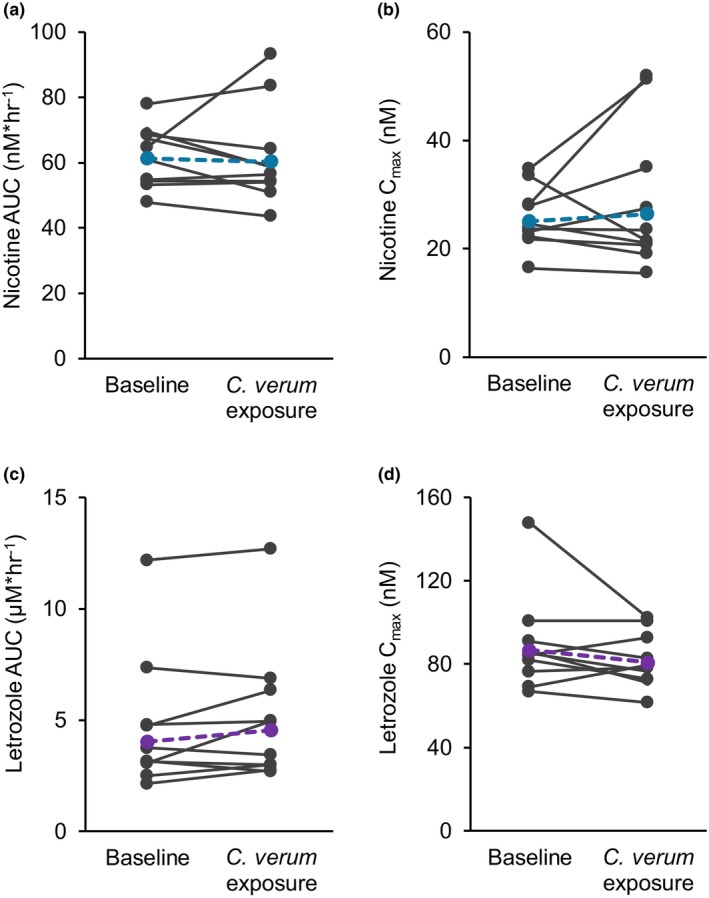
Individual nicotine (**a**) AUC (*n* = 10) and (**b**) *C*_max_ (*n* = 10) and letrozole (**c**) AUC (*n* = 10) and (**d**) *C*_max_ (*n* = 10) at baseline and after exposure to *C. verum* (2 g) thrice daily for 6 consecutive days. Colored circles and dashed lines denote geometric means.

The geometric mean plasma concentration *vs*. time profile for both nicotine and letrozole was nearly superimposable in the presence vs. absence of *C. verum* (**Figure** **1**). Correspondingly, the geometric mean ratios for *C*_max_, AUC, t_1/2_, and CL/F for both drugs were near unity (**Table** **1**). Except for cotinine and 3‐hydroxycotinine AUC and cotinine *C*_max_, corresponding metrics for cotinine and 3‐hydroxycotinine could not be determined due to insufficient data points to obtain a robust estimate of the terminal slope (**Table** **1**). Nicotine and letrozole AUC and *C*_max_ for each participant are displayed as spaghetti plots (**Figure** **2**).

**Table 1:** Pharmacokinetics of nicotine, cotinine, 3‐hydroxycotinine, and letrozole in the presence and absence of *Cinnamomum verum*

	*C*_max_ (nM)	AUC (h*nM)	*t*_max_ (h)	*t*_1/2_ (h)	CL/F (L/h)
Nicotine					
Baseline (*n* = 10)	23 (20–27)	61 (56–67)^a^	0.5 (0.5–1.5)	3.4 (2.9–3.9)	111 (95–130)
*C. verum* exposure (*n* = 10)	25 (21–31)	60 (53–69)^a^	0.5 (0.5–1.5)	3.6 (3.1–4.3)	108 (94–123)
*C. verum e*xposure/Baseline	1.10 (0.93–1.26)	0.98 (0.91–1.07)	–	1.10 (1.04–1.16)	0.96 (0.88–1.05)
Cotinine					
Baseline (*n* = 11)	64 (51–81)	555 (472–653)^b^	4 (3–8)	–	–
*C. verum* exposure (*n* = 11)	54 (46–64)	530 (452–621)^b^	4 (3–6)	–	–
*C. verum e*xposure/Baseline	0.85 (0.68–1.05)	0.95 (0.85–1.07)	–	–	–
3‐Hydroxycotinine					
Baseline (*n* = 11)	–	105 (76–146)^b^	–	–	–
*C. verum* exposure (*n* = 11)	–	94 (71–123)^b^	–	–	–
*C. verum e*xposure/Baseline	–	0.89 (0.74–1.08)	–	–	–
Letrozole					
Baseline (*n* = 10)	87 (76–99)	4060 (3000–5510)^c^	1.5 (1.0–3.0)	46 (36–60)	2.16 (1.59–2.92)
*C. verum* exposure (*n* = 10)	81 (74–89)	4400 (3160–6130)^c^	2.0 (1.0–6.0)	49 (37–64)	1.98 (1.42–2.77)
*C. verum e*xposure/Baseline	0.93 (0.86–1.02)	1.10 (0.98–1.24)	–	1.05 (0.97–1.13)	0.90 (0.79–1.03)

Values denote geometric means (90% confidence intervals) except *t*_max_, which denotes median (min‐max) (*P* > 0.16 for all comparisons, paired Student's *t*‐test). *C*_max_, maximum concentration; AUC, area under the plasma concentration vs. time curve; *t*_max_, time to reach *C*_max_; *t*_1/2_, terminal half‐life; CL/F, apparent clearance.

^a^ AUC from 0 to 4 hours.

^b^ AUC from 0 to 12 hours.

^c^ AUC from time zero to infinity.

**Figure 3:**
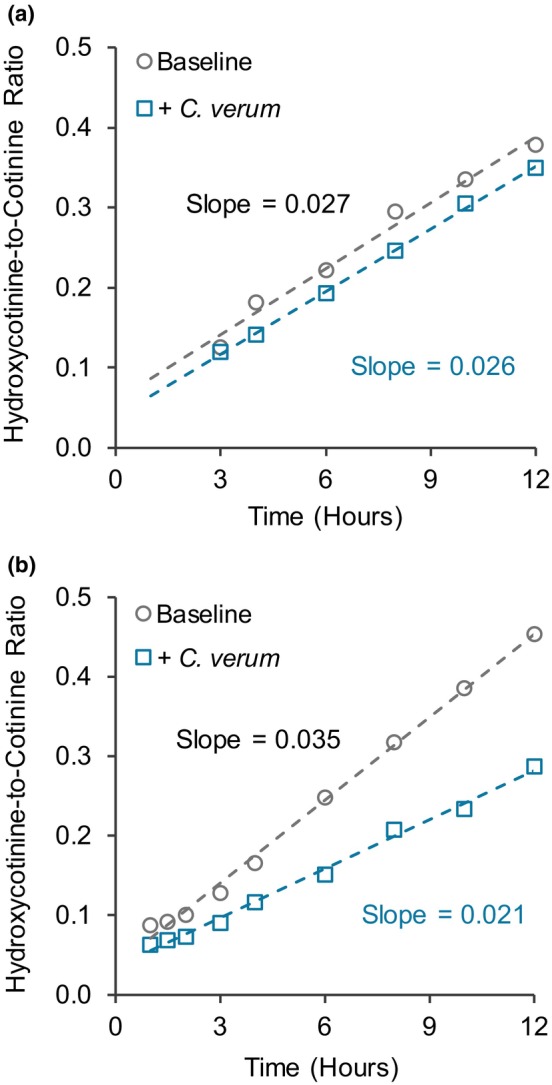
Representative nicotine metabolite ratio vs. time plots showing the rate of metabolite formation (slope) at baseline and after exposure to *C. verum*. Panel **a** shows no change in slope in the presence of *C. verum*, suggesting no inhibition or the inhibitor was metabolized. Panel **b** shows a decrease in the slope in the presence of *C. verum*, suggesting inhibition occurred with time.

**Figure 4:**
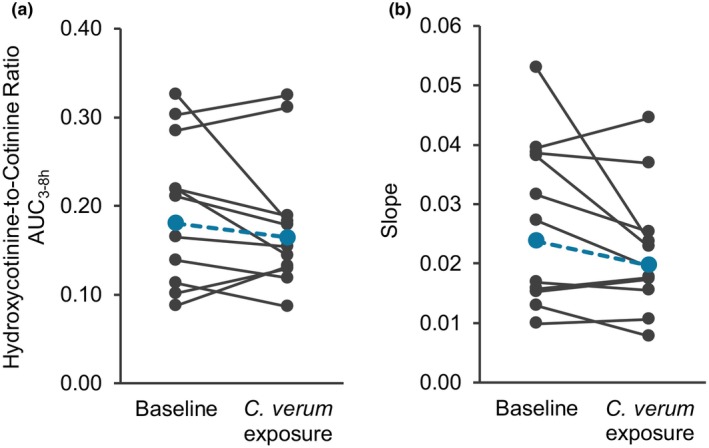
Individual nicotine metabolite ratio AUC_3−8h_ (left, *n* = 11) and slope of nicotine metabolite ratio vs. time (right, *n* = 11) at baseline and after exposure to *C. verum*. Colored circles and dashed lines denote geometric means.

The GMR for the NMR at all three selected time points and AUC_3−8h_ was ~0.90 (**Table** **2**), indicating no interactions. A notable observation was that the NMR increased proportionally with time. Accordingly, the slope approach was used to further evaluate *C. verum*‐mediated CYP2A6 inhibition by characterizing the NMR as a function of time (the slope) (**Figure** **3**). The GMR for the slope was 0.83 [0.70–0.98] (**Table** **2**). AUC_3−8h_ of the NMR vs. time plot and the slope for each participant are displayed as spaghetti plots (**Figure** **4**).

**Table 2:** Nicotine metabolite ratio

	Baseline (*n* = 11)	*C. verum* exposure (*n* = 11)	*Cinnamomum verum* exposure/Baseline
Time (hours)	3‐Hydroxycotinine‐to‐cotinine ratio		
4	0.15 (0.12–0.19)	0.13 (0.11–0.16)	0.90 (0.77–1.05)
6	0.20 (0.15–0.25)	0.18 (0.14–0.22)	0.91 (0.77–1.06)
8	0.24 (0.18–0.31)	0.21 (0.17–0.27)	0.90 (0.76–1.07)
AUC_3–8h_	0.18 (0.14–0.23)	0.16 (0.13–0.20)	0.91 (0.78–1.07)
	Slope (hour^−1^)		
All time points	0.022 (0.016–0.030)	0.019 (0.015–0.025)	0.87 (0.70–1.09)
Partial linear time points	0.024 (0.018–0.032)	0.020 (0.015–0.026)	0.83 (0.70–0.98)

Values denote geometric means (90% confidence intervals) (*P* > 0.076 for all comparisons, paired Student's *t*‐test).

## DISCUSSION

Cinnamon has a long‐established history as a flavoring agent and, more recently, as a botanical dietary supplement that is promoted for various health‐supporting properties. *C. verum* is particularly popular among the different *Cinnamomum* species due to purported glucose‐lowering effects.^23^ Previous mechanistic in vitro studies demonstrated that both cinnamaldehyde and methoxycinnamaldehyde could increase the AUC—up to 5‐fold—of the CYP2A6 substrates nicotine and letrozole,^7^, ^8^ prompting the current clinical study. Although results indicated no interactions, a new tool, termed the slope approach, was applied to the nicotine data that shed new insight into the time‐dependent effects of the *C. verum* product on CYP2A6 activity.

The pharmacokinetic profiles of both nicotine gum and letrozole at baseline were consistent with previous studies.^20^, ^24^, ^25^, ^26^, ^27^, ^28^ The predicted 5‐fold increase in nicotine AUC^7^, ^8^ in the presence of cinnamaldehyde assumed a cinnamaldehyde dose of 275 mg, which is the maximum reported dose of cinnamon powder (up to 10 g) consumed by patients with diabetes. Applying the same mechanistic static model using the cinnamaldehyde dose (17 mg) contained in a single 2‐gram dose of *C. verum* used in the current study, the AUC of both nicotine and letrozole was predicted to increase by 1.3‐fold, which still exceeds the cut‐off. Applying an accumulation ratio [1/(1‐e^‒kτ^)] of 1.8, calculated using the reported half‐life for cinnamaldehyde in rats (6.7 h) and a dosing interval (τ) of 8 h, the AUC of both substrates was predicted to increase by 1.5‐fold upon multiple dosing of *C. verum* for five consecutive days. The trace amount of methoxycinnamaldehyde in the *C. verum* product precluded a sound prediction of the effects of methoxycinnamaldehyde on nicotine and letrozole AUC. In contrast to these predictions, there were no effects of *C. verum* on various pharmacokinetic endpoints (**Table** **1**), which were likely due to extensive elimination, both first‐pass and systemic, of cinnamaldehyde, leading to insufficient concentrations in the liver to effectively inhibit CYP2A6. Although the pharmacokinetics of cinnamaldehyde have not been determined in humans (reasons detailed later), studies in animal models, particularly in rats, support this notion.^29^, ^30^

Cinnamaldehyde was shown to be unstable in the intestine after oral administration to rats. Specifically, the reactive aldehyde group readily underwent oxidation to form cinnamic acid, predominantly by aldehyde dehydrogenase.^31^ After intravenous administration, cinnamaldehyde was extensively distributed to the tissues, including the liver, brain, heart, and kidneys, where aldehyde dehydrogenation also occurs.^30^, ^32^, ^33^ Urine data further suggested that cinnamaldehyde is converted to hippuric acid and benzoic acid through beta‐oxidation.^29^, ^30^, ^31^, ^34^ Besides oxidation, cinnamaldehyde can be reduced to cinnamyl alcohol in the intestine and stomach.^32^ Despite species differences in drug metabolism being well recognized, cinnamaldehyde metabolism involves multiple pathways and occurs in multiple tissues. Collectively, these processes likely contribute to the presumed low oral bioavailability and extensive elimination of cinnamaldehyde, resulting in ineffective hepatic CYP2A6 inhibitory concentrations in the clinical study participants.

A low extent of cinnamaldehyde absorption would also impact oral bioavailability and, in turn, hepatic cinnamaldehyde exposure and CYP2A6 inhibition by *C. verum*. The solubility, dissolution rate, and permeability of cinnamaldehyde have not been thoroughly evaluated. Assuming a logP of 1.98 and a low aqueous solubility of 1.42 mg/mL (PubChem CID 637511), a 17‐mg dose of cinnamaldehyde is predicted to be moderately soluble in 250 mL of intestinal fluid (0.07 mg/mL). These predictions are consistent with a recent study reporting a “bioaccessibility” of cinnamaldehyde of ~0.30 mg/mL in simulated human gastric and intestinal fluids.^35^ The extensive distribution of cinnamaldehyde in rats further suggests that cinnamaldehyde permeates tissues.^30^, ^32^ Thus, the presumed low extent of cinnamaldehyde absorption may largely reflect a slow dissolution rate. Further research is needed to recover the absorption parameters and potentially develop a physiologically based pharmacokinetic model to gain additional mechanistic insight into cinnamaldehyde absorption.

One arm of the current study included administering a single dose of *C. verum* to six healthy adults to characterize the pharmacokinetics of cinnamaldehyde and potentially other constituents. However, attempts to quantify cinnamaldehyde in plasma by UPLC‐MS/MS were unsuccessful due to the instability of cinnamaldehyde, potential rapid oxidation under air exposure, and presumed low oral bioavailability (discussed above). Bioanalysis using gas chromatography coupled with mass spectrometry (GC–MS) has been reported in rat studies.^30^, ^32^ The estimated systemic concentration (up to 52 nM) from a 17‐mg cinnamaldehyde dose used in the current study was below the LOQ of ~150 nM. Our attempt to analyze cinnamaldehyde using a GC–MS method confirmed a similar LOQ (data not shown). Collectively, plasma cinnamaldehyde concentrations upon both single and multiple *C. verum* doses were likely in the non‐quantifiable range. A higher dose of cinnamaldehyde may produce plasma concentrations above the LOQ, allowing pharmacokinetic characterization of cinnamaldehyde. The high safety profile of cinnamaldehyde, as evidenced by a NOAEL (no observed adverse effect level) equivalent to 125 mg/kg in mice^36^ that translates to a human equivalent dose of 10.1 mg/kg, indicates such studies are feasible.

The nicotine metabolite ratio (NMR) is used to determine CYP2A6 activity, primarily to phenotype individuals for smoking behavior and develop personalized cessation therapies.^37^, ^38^ Due to the complex metabolism of nicotine,^39^ along with *CYP2A6* being highly polymorphic,^40^, ^41^, ^42^, ^43^ a secondary analysis of the NMR data was conducted to further assess the effects of *C. verum* on CYP2A6 activity. Results indicated that *C. verum* could inhibit CYP2A6 activity in some individuals, by up to ~20% (**Table** **2**). Representative NMR vs. time profiles (**Figure** **S3**) showed fluctuations between baseline and *C. verum* exposure at the different time points, raising concern about the reliability of using a single time point to evaluate potential CYP2A6‐mediated drug interactions. Although AUC of the NMR vs. time data minimizes fluctuations and provides a better measure of enzyme activity, temporal changes in activity cannot be assessed. Accordingly, an additional empirical approach involving the slope of the NMR vs. time profile (the “slope approach,” which reflects the rate of change in CYP2A6 activity) was applied to provide a dynamic assessment of the inhibitory effect of *C. verum* on CYP2A6 activity while minimizing variability in the NMR at different time points. Because *C. verum* was expected to decrease both the NMR and the slope, two mechanistic interpretations for different scenarios can be inferred. First, if the slope for *C. verum* exposure was parallel with that at baseline, then CYP2A6 activity was not inhibited, at least in a reversible manner; alternatively, cinnamaldehyde was metabolized, allowing enzyme function to return to baseline due to time‐dependent inhibition (**Figure** **3** **a**). Second, if the slope for *C. verum* exposure was lower than that at baseline, then CYP2A6 activity was inhibited in a time‐dependent manner (**Figure** **3** **b**). Results from approximately half of the participants aligned with either scenario (**Figure** **4** **b**).

Application of the metabolite‐to‐substrate ratio combined with the slope approach may extend beyond drug interaction studies involving nicotine. As shown in the current work, addition of the slope approach provided further insight into the mechanism of CYP2A6 inhibition in each participant. Potential benefits of this combined approach include (1) reducing inter‐individual variability in pharmacokinetic endpoints, (2) assessing enzyme inhibition mechanisms, and (3) “normalizing” the effects of genetic polymorphisms.

Our proposed slope approach has limitations. First, the insignificant changes in nicotine pharmacokinetics may have precluded validation, particularly due to the small sample size. Some participants were excluded from the analysis due to analyte concentrations being below the LOQ or prior exposure to nicotine on a study day. Second, a slope will only be observed during the formation phase of the metabolite or when the metabolite half‐life is longer than that of the parent (i.e., when the metabolite follows elimination rate‐limited kinetics). Distribution and first‐pass metabolism could also impact observation of a slope. Third, although there are benefits to applying a new analysis tool to clinical drug interaction studies, the slope approach lacks practicality in most clinical settings. Considering cost and patient compliance, determining NMR at one time point remains the preferred method, especially for phenotyping individuals who use nicotine to tailor therapy more effectively. Therefore, while the slope approach is not recommended for treatment implementation of nicotine and potentially other drugs, it may serve as a valuable analytical tool for evaluating clinical drug interaction studies in general. Future pharmacokinetic studies involving a larger sample size, higher dose of cinnamaldehyde, and potent CYP2A6 inhibitor (e.g., methoxsalen or selegiline) could help address these limitations.

In summary, a typical dose of a well‐characterized *C. verum* product showed no interactions with two clinically relevant CYP2A6 substrate drugs in healthy adults. The general lack of effect of *C. verum* on the pharmacokinetics of the CYP2A6 substrates nicotine and letrozole suggests that *C. verum* may be safe to consume with both drugs and other CYP2A6 substrates. A novel analysis tool using NMR at multiple time points, termed the slope approach, was applied to gain further mechanistic insight into CYP2A6 inhibition. This approach may have applications to future enzyme‐mediated drug interaction studies.

## FUNDING

This work was supported by the National Center for Complementary and Integrative Health and the Office of Dietary Supplements (U54 AT008909); Bethesda, MD.

## CONFLICT OF INTEREST

The authors declared no competing interests for this work.

## AUTHOR CONTRIBUTIONS

A‐H.P.N and M.F.P. – wrote the manuscript; D.L.H., D.A.T., J.R.W., M.E.L., N.B.C., K.E.T., M.F.P. – designed the research; A‐H.P.N., D.L.H., D.A.T., P.K.M., N.B.C., K.E.T. – performed the research; A‐H.P.N, D.A.T., P.K.M., and M.F.P. – analyzed the data.

## Supporting information


Data S1.
