# Supplementary material for: Pharmacokinetic Evaluation of a Cinnamon Product on CYP2A6 Substrate Drugs: Application of a Novel Tool Involving the Nicotine Metabolite Ratio
Source: Clin Pharmacol Ther. 2026 Feb 1;119(6):1485–93. doi: 10.1002/cpt.70218 (PMC13156345; doi:10.1002/cpt.70218)
Supplement: Supplementary file 1 — Data S1. [file CPT-119-1485-s001.pdf]

Supplemental information

Pharmacokinetic Evaluation of a Cinnamon Product on CYP2A6 Substrate Drugs: Application of a Novel Tool Involving the Nicotine Metabolite Ratio

Aiden-Hung P. Nguyen[1], Deena L. Hadi[1,2], Daniel A. Todd[3], Preston K. Manwill[3], John R. White[1], Matthew E. Layton[5], Nadja B. Cech[2,3], Kenneth E. Thummel[2,4], Mary F. Paine[1,2]

[1]College of Pharmacy and Pharmaceutical Sciences, Washington State University, Spokane, WA, USA
[2]Center of Excellence for Natural Product Drug Interaction Research, Spokane, WA, USA
[3]Department of Chemistry and Biochemistry, University of North Carolina at Greensboro, Greensboro, NC, USA
[4]Department of Pharmaceutics, School of Pharmacy, University of Washington, Seattle, WA, USA
[5]Elson S. Floyd College of Medicine, Washington State University, Spokane, WA, USA

Corresponding author: Mary F. Paine, 412 E Spokane Falls Blvd, Spokane, WA, 99202, USA, mary.paine@wsu.edu

Methods for cinnamon product analysis (C83)

1. Cinnamon product selection

The cinnamon product (arbitrarily coded as C83) [500 mg *Cinnamomum verum* (Ceylon cinnamon) bark powder per capsule, Ceylon Cinnamon Shop, Issaquah, WA] was selected from a set of 60 commercial cinnamon supplements according to best practices for botanical natural products research.[1] Ultra-performance liquid chromatography-mass spectrometry (UPLC-MS) analyses were used to compare the composition of this product to authentic Ceylon cinnamon (*Cinnamomum verum* J. Presl.) and other commercially available cinnamon supplements.[2–5]

2. Metabolite identification and quantification

2.1. Materials and chemicals

The *trans*-cinnamaldehyde (CAS # 14371-10-9) standard was purchased from Alfa Aesar (Ward Hill, MA), the 2-methoxycinnamaldehyde (CAS # 1504-74-1) standard was purchase from Santa Cruz Biotechnology (Dallas, TX), and the coumarin (CAS # 91-64-5) standard was purchased from Tokyo Chemical Industry (Portland, OR).

2.2. Preparation of calibration standards

Primary stock solutions of analytes were prepared separately by dissolving an accurately weighed quantity of each standard in an appropriate volume of UHPLC-MS-grade methanol to achieve a concentration of 10 mM. A combined stock of the analytes was prepared with the concentration of each constituent at 100 µM in methanol. The combined stock was then serially diluted two-fold to produce a dilution series ranging from 8 – 100000 nM.

2.3. Instrumentation and analytical conditions

Chromatographic analysis of the metabolites was conducted utilizing a Waters Acquity Ultra-Performance Liquid Chromatography (UPLC) system (Waters, Milford, MA, USA) coupled to a Q Exactive Plus mass spectrometer (Thermo Fisher Scientific, Waltham, MA). System was operated using Thermo Scientific™ Xcalibur™ software version 3.0 (Thermo Fisher Scientific, Waltham, MA). UPLC system consisted of the following modules: a sample manager, photodiode array detector (PDA), column manager, and binary solvent manager. Injections of 3 µL were performed on an Acquity UPLC HSS T3 column (Waters, 2.1 × 100 mm, 1.8 µm) operated with a flow rate of 0.5 mL/min and a column temperature of 40 °C. The mobile phase consisted of 0.01% formic acid in water (A) and 0.01% formic acid in acetonitrile (B). The starting gradient conditions of 95% A and 5% B were held for 0.3 min, followed by a linear increase in solvent B from 5% to 90% over 8.8 min, a linear increase in solvent B from 90% to 98% over 1.6 min, an isocratic hold at 100% for 0.3 min, a sharp decrease to the starting conditions of 5% B over 0.1 min, and finished with a 1.7 min isocratic hold. The mass spectrometer was equipped with a heated electrospray ionization (HESI) source operating with the following parameters: spray voltage of 3.5 kV, vaporizer temperature of 438 °C, capillary temperature of 269 °C, S-lens RF level of 50, sheath gas, auxiliary gas, and sweep gas of 43, 14, and 3 (arbitrary units), respectively. Nitrogen was used as the source gas.

Detection of the metabolites was achieved using the PDA detector with a range from 250 to 300 nm (**Figure S1**). Metabolites were simultaneously detected via high-resolution MS (HRMS) in positive ionization mode using a full scan MS experiment with a mass range from 100 to 1500 *m/z*, a resolution of 35,000, an AGC target of 1E6, and a maximum injection time (IT) of 50 ms.

The spectrometer was calibrated weekly using Thermo Scientific™ Pierce™ LTQ Velos ESI Positive Ion Calibration Solution (Thermo Fisher Scientific, Waltham, MA).

Data acquisition and quantitative analysis of the analytes were accomplished using Thermo Scientific™ Xcalibur™ software version 3.0 (Thermo Fisher Scientific, Waltham, MA). Peak detection and peak areas using the UV data were determined using the Genesis algorithm. All calibration curves were generated in Xcalibur™ using $1/X^2$ weighting to create linear curve fits that emphasize the lower concentration calibration points (**Table S1**). External calibration was employed for this analysis.

2.4. Preparation and UPLC-MS analysis of commercial cinnamon product

Triplicate extractions of the cinnamon product (C83) were conducted by adding 200 mg of powdered cinnamon material and 20 mL of reagent-grade methanol to a 20 mL scintillation vial. The mixtures were shaken 24 h at 20 °C and 150 rpm, filtered, and dried under nitrogen. The dried methanolic extracts of the commercial product were reconstituted to 5 mg/mL in an appropriate volume of Optima® UPLC-MS grade methanol (Fisher Scientific, Waltham, MA, USA). All samples (100 µL) were diluted with 900 µL of Optima® UPLC-MS grade methanol, followed by another 1 to 10 dilution with Optima® UPLC-MS grade methanol. Samples were analyzed at 5 µg/mL and 50 µg/mL with freshly prepared calibration standards using the UPLC-PDA-HRMS method described above.

References

1. Kellogg, J. J., Paine, M. F., McCune, J. S., Oberlies, N. H. & Cech, N. B. Selection and characterization of botanical natural products for research studies: a NaPDI center recommended approach. *Nat Prod Rep* **36**, 1196–1221 (2019).
2. Wallace, E. D., Oberlies, N. H., Cech, N. B. & Kellogg, J. J. Detection of adulteration in Hydrastis canadensis (goldenseal) dietary supplements via untargeted mass spectrometry-based metabolomics. *Food Chem Toxicol* **120**, 439–447 (2018).
3. Wallace, E. D., Todd, D. A., Harnly, J. M., Cech, N. B. & Kellogg, J. J. Identification of adulteration in botanical samples with untargeted metabolomics. *Anal Bioanal Chem* **412**, 4273–4286 (2020).
4. Kellogg, J. J., Kvalheim, O. M. & Cech, N. B. Composite score analysis for unsupervised comparison and network visualization of metabolomics data. *Anal Chim Acta* **1095**, 38–47 (2020).
5. Nguyen, J. T. *et al.* Assessing Transporter-Mediated Natural Product-Drug Interactions Via In vitro-In Vivo Extrapolation: Clinical Evaluation With a Probe Cocktail. *Clin Pharmacol Ther* **109**, 1342–1352 (2021).

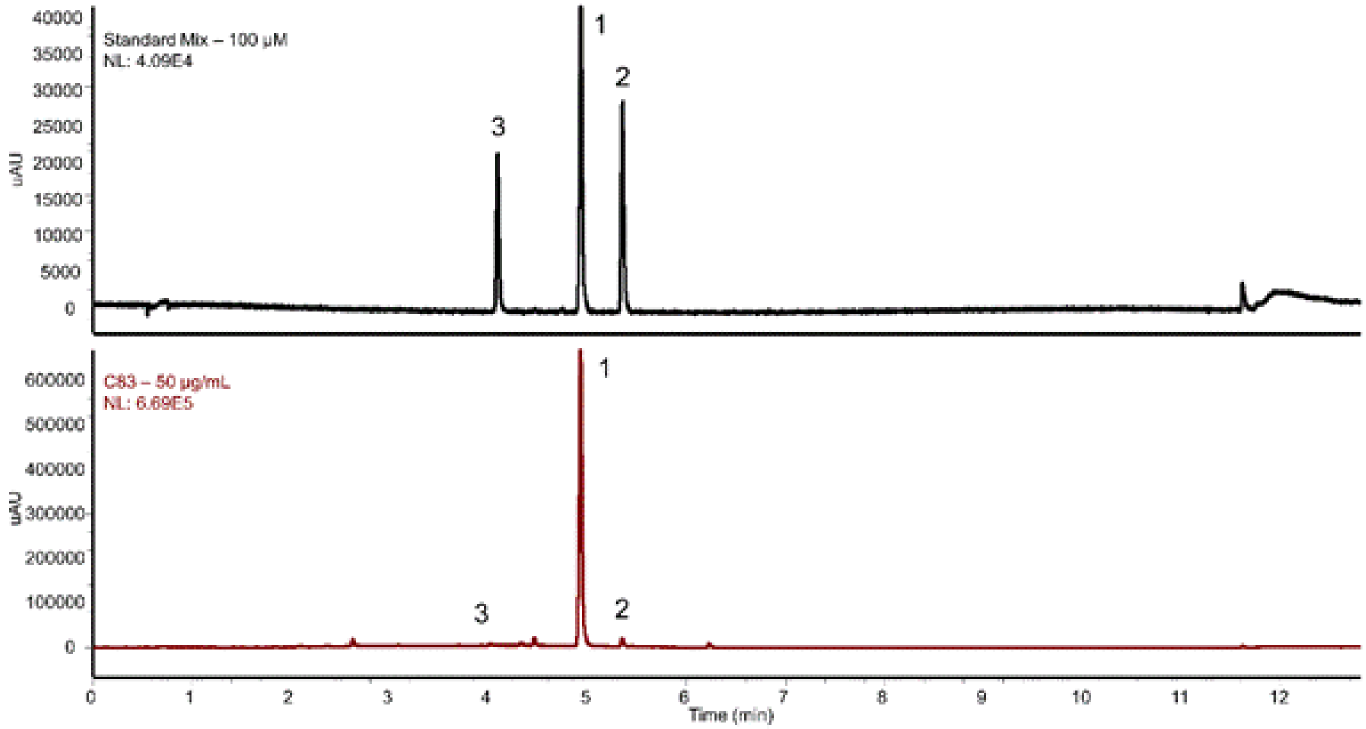

Figure S1. UPLC-MS chromatograms for the cinnamon product (Ceylon Cinnamon Shop, C83) at 50 µg/mL (bottom) and a standard mixture of trans-cinnamaldehyde (1), 2-methoxycinnamaldehyde (2), and coumarin (3) at 100 µM (top).

Table S1. Concentrations of constituents in cinnamon (*Cinnamomum verum*) bark powder supplement (C83) and extract prepared from C83.

Constituents	Structure	Extract Concentration [a] (mg/g)	Concentration in supplement [b] (mg/g)	Quantity in a single 500 mg capsule (mg)
trans-cinnamaldehyde	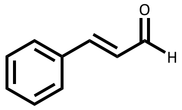	83.1 ± 9.0	8.27 ± 0.90	4.135 ± 0.45
2-methoxycinnamaldehyde	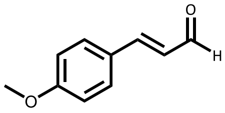	5.52 ± 0.53	0.549 ± 0.053	0.275± 0.027
coumarin	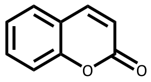	0.260 ± 0.014	0.0259 ± 0.0014	0.0130± 0.0007

All values denote means ± standard deviations for three separate extractions of the cinnamon material from the C83 supplement, each analyzed independently. [a]Mean mass of compound per mg of dried residue of methanolic extract of C83. [b]Mean mass of compound per mass of cinnamon bark powder from the capsule.

Table S2. Study inclusion and exclusion criteria

Inclusion
• Males and non-pregnant, non-lactating females aged from 18-64 years and healthy<br>• Not taking any medications (prescription and non-prescription) or dietary/herbal supplements known to alter the pharmacokinetics of either study drug or cinnamon constituents<br>• Willing to abstain from consuming dietary/herbal supplements and citrus juices for several weeks<br>• Willing to abstain from consuming caffeinated beverages or other caffeine-containing products the evening before and morning of the first day of a study arm<br>• Willing to abstain from consuming any alcoholic beverages for one day prior to any study day, during the 14-hour inpatient days, and for the outpatient visit(s) following the 14-hour visit<br>• Willing to use an acceptable method of contraception that does not include oral contraceptive pills or patches (such as abstinence, copper IUD, or condom)<br>• Have the time to participate<br>• Written informed consent (and assent when applicable) obtained from subject or subject's legal representative and the ability for the subject to comply with the requirements of the study
Exclusion
• Under the age of 18 or 65+ years<br>• Any current major illness or chronic illness such as (but not limited to) kidney disease, hepatic disease, diabetes mellitus, hypertension, coronary artery disease, chronic obstructive pulmonary disease, cancer, or HIV/AIDS<br>• History of anemia or any other significant hematologic disorder<br>• History of drug or alcohol addiction or major psychiatric illness<br>• Pregnant or nursing<br>• History of allergy to cinnamon, letrozole, or nicotine<br>• Taking concomitant medications, both prescription and non-prescription (including dietary supplements/herbal products), known to alter the pharmacokinetics of either study drug or cinnamon constituents<br>• Presence of a condition or abnormality that, in the opinion of the Investigator, would compromise the safety of the patient or the quality of the data<br>• Recreational drug use such as amphetamines, benzodiazepines, cocaine, marijuana, MDMA, opioids, and PCP<br>• History of intolerance to cinnamon<br>• Out-of-range clinical laboratory value that the study physician considers participation in the study a health risk

Table S3. Bioanalytical procedures.

Analyte	Nicotine (NIC)	Cotinine (COT)	3-Hydroxycotinine (3HC)	Letrozole (LTZ)
System	Agilent 1290 UPLC coupled with Agilent 6410 triple-quadrupole			AB SciEx 6500 Q-TRAP
LC column	HyperCarb 1.8 µm, 100 mm x 2.1 mm			ACQUITY UPLC HSS T3 1.8 µm, 2.1x100 mm
Column temperature (°C)	40°C			40°C
Mobile phase A	20 mM ammonium acetate, pH 10			0.1% FA in water
Mobile phase B, gradient (min)	Methanol<br>10% B (5),<br>→ 70% B (15)<br>→ 70% B (16)<br>→ 10% B (16.1)<br>→ 10% B (18)			0.1% FA in acetonitrile<br>15% B (0.1)<br>→ 90% B (0.75)<br>→ 90% B (2.5)<br>→ 15% B (2.6)<br>→ 15% B (3.5)
Ion mode	Positive			Negative
Internal standard	d_4-NIC	d_3-COT	d_3-3HC	d_4-LTZ
Transition (m/z)	NIC: 163.1 > 83<br>d_4-NIC: 167 > 84	COT: 177.1 > 80.1<br>d_3-COT: 180.1 > 80.1	3HC: 193.1 > 80.1<br>d_3-3HC: 196 > 80.1	LTZ: 284 > 242<br>d_4-LTZ: 288 > 246
Declustering potential (V)	--	--	--	LTZ: -5 V<br>d_4-LTZ: -100 V
Fragmentor voltage (V)	NIC: 75 V<br>d_4-NIC: 75 V	COT: 96 V<br>d_3-COT: 111 V	3HC: 111 V<br>d_3-3HC: 111 V	--
Collision energy (V)	NIC: 20 V<br>d_4-NIC: 22 V	COT: 28 V<br>d_3-COT: 28 V	COT: 32 V<br>d_3-COT: 30 V	LTZ: -34 V<br>d_4-LTZ: -36 V
Limit of quantitation	1.5 nM	1.5 nM	1.5 nM	1.56 nM

FA, formic acid

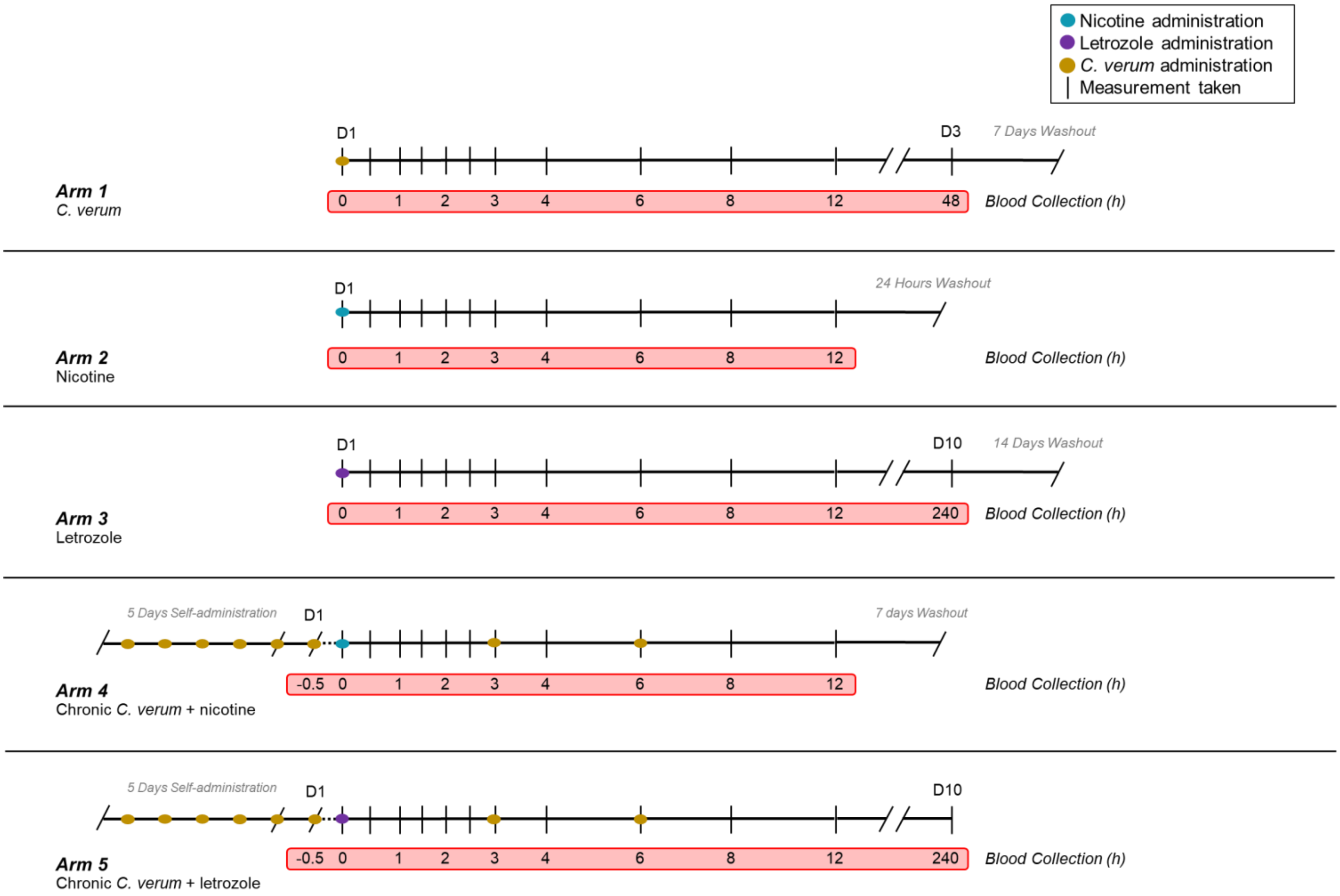

Figure S2. Clinical study design. Healthy adults were enrolled in a crossover, open-label, fixed-sequence study according to the inclusion/exclusion criteria. Arms 1, 2, and 3 (baseline) entailed the administration of C. verum (2 g), nicotine gum (2.5 mg), and a letrozole tablet (2.5 mg), respectively. After a washout period of at least 7 days, 24 hours, and 14 days, respectively, participants self-administered the C. verum product (2 g) thrice daily for 5 consecutive days. On day 6, they were administered nicotine gum (Arm 4) or letrozole (Arm 5) 30 minutes after taking C. verum; two additional doses of C. verum (2 g) were administered in 4-hour increments. During all Arms, plasma was collected at designated times (shaded in red) up to 12 hours and 240 hours post-nicotine and -letrozole administration, respectively

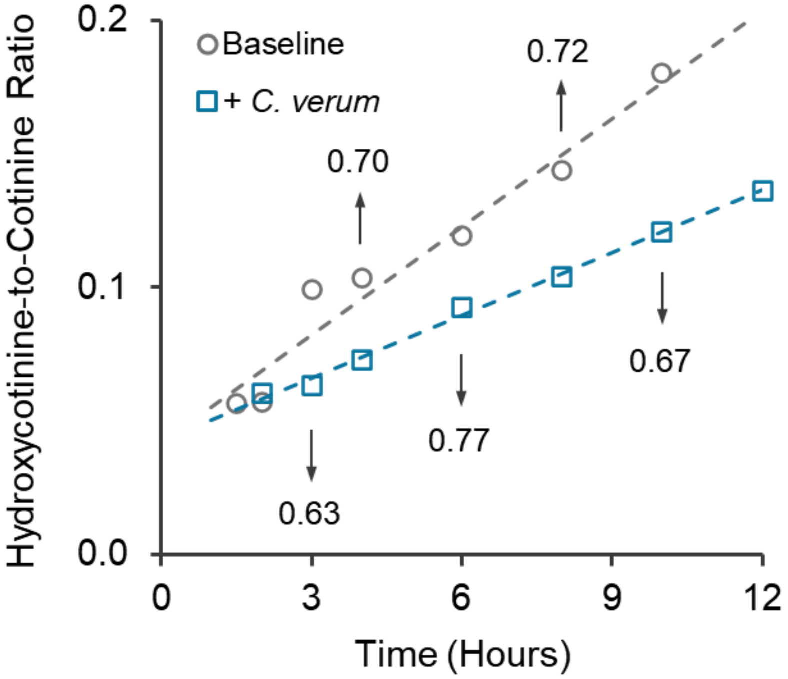

Figure S3. A representative nicotine metabolite ratio plot showing varying ratios of C. verum exposure to baseline at different time points, which is indicated by the arrow and number on the plot.

Certificate of Analysis – Ceylon cinnamon

Certificate of Analysis

Botanical: *Cinnamomum verum* (Ceylon cinnamon)

Product name: Organic Ceylon Cinnamon Capsules, 500 mg per capsule, Bark

Manufacturer/Supplier: Ceylon Cinnamon Shop

Lot number: 757120

NaPDI code: C83

Date analyzed: 06/17/2021

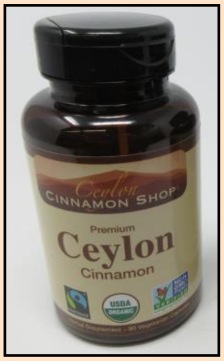

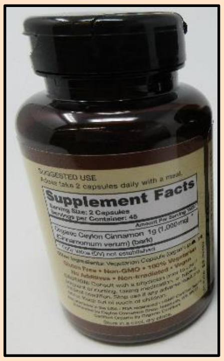

Quantified Metabolites

Metabolite	Quantity (mg/g)
1. cinnamaldehyde	8.27 ± 0.90
2. 2-methoxycinnamaldehyde	0.549 ± 0.053
3. coumarin	0.0259 ± 0.0014

*Amount is expressed as milligrams of compound per gram of powdered product.

Contamination Analyses

Heavy metals	Passed
Pesticides	Passed
Residual solvents	Passed
Microbial contamination	Passed

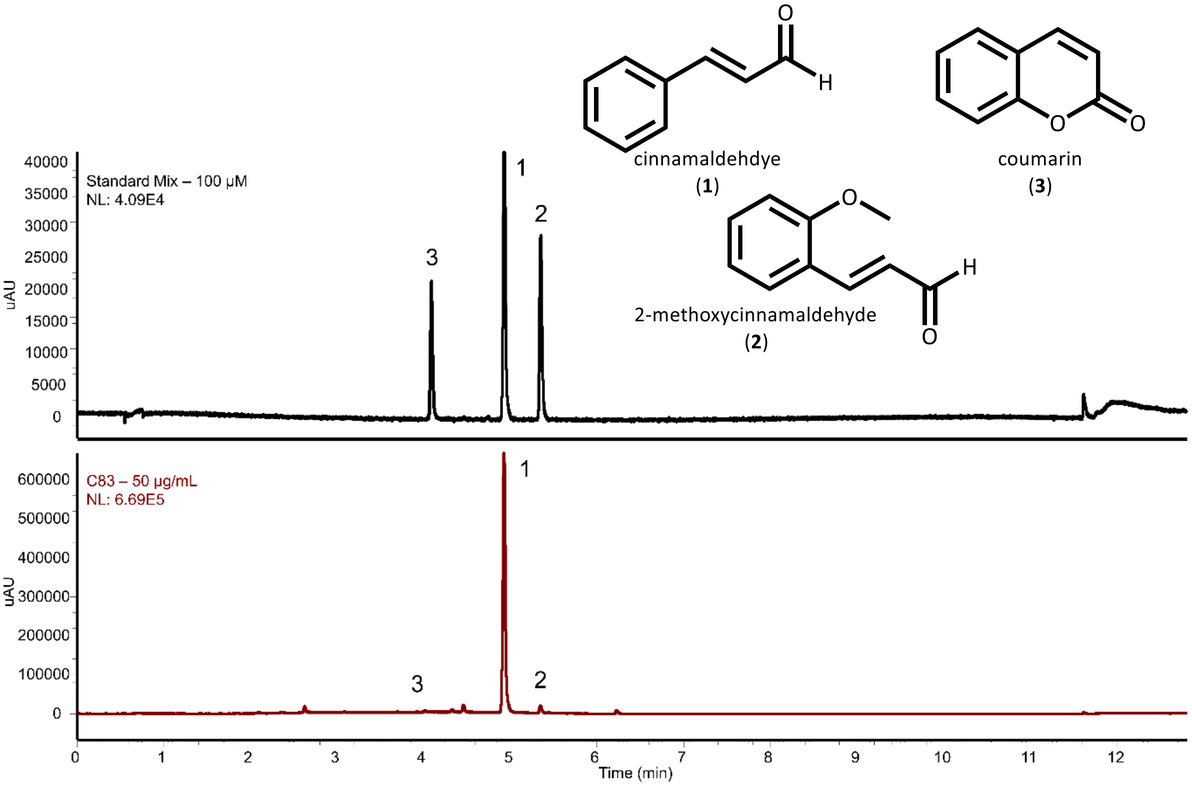

Certificate of Analysis

Botanical: *Cinnamomum verum* (Ceylon cinnamon)

Product name: Organic Ceylon Cinnamon Capsules, 500 mg per capsule, Bark

Manufacturer/Supplier: Ceylon Cinnamon Shop

Lot number: 757120

NaPDI code: C83

Date analyzed: 06/17/2021

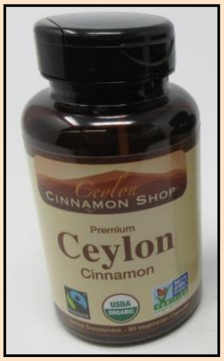

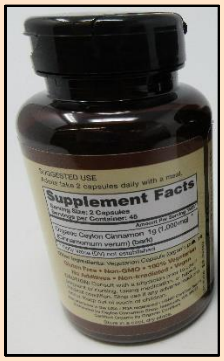

Metabolite Test Results

See page 4 for details.

Metabolite	Quantity (mg/g)
1. cinnamaldehyde	8.27 ± 0.90
2. 2-methoxycinnamaldehyde	0.549 ± 0.053
3. coumarin	0.0259 ± 0.0014

*Amount is expressed as milligrams of compound per gram of powdered product.

Heavy Metals Test Results

AOAC 2011.19 and 993.14 (modified), ISO/IEC 17025:2017 A2LA 2918.01 (Eurofins)

Heavy Metal	Quantity	Allowable
Arsenic	<10.0 ppb	10 ppm
Cadmium	57.7 ppb	4.1 ppm
Lead	73.9 ppb	10 ppm
Mercury	<5.00 ppb	2.0 ppm

*Allowable limit established by American Herbal Products Association Guidelines.

Pesticides Test Results

GC-MS/MS – AOAC 2007.01, ISO/IEC 17025:2017 A2LA 2993.01; LC-MS/MS – AOAC 207.01, ISO/IEC 17025:2017 A2LA 2993.01 (Eurofins)

Pesticides	Quantity
Screened pesticides	Not Detected at LOQ

Residual Solvents Test Results

Class I – USP/NF 467 (modified)(Eurofins)

Residual solvent	Quantity	Allowable
Benzene	< 2 ppm	2 ppm
Carbon tetrachloride	< 4 ppm	4 ppm
1,2-dichloroethane	< 5 ppm	5 ppm
1,1-dichloroethene	< 3 ppm	8 ppm
1,1,1-trichloroethane	< 10 ppm	1500 ppm

Class II – USP/NF 467 (Modified)(Eurofins)

Residual solvent	Quantity	Allowable
Acetonitrile	< 50 ppm	410 ppm
Chlorobenzene	< 5 ppm	360 ppm
Chloroform	< 5 ppm	60 ppm
Cumene	< 5 ppm	N/A
Cyclohexane	< 5 ppm	3880 ppm
1,2-dichloroethene	< 5 ppm	1870 ppm
1,2-dimethoxyethane	< 20 ppm	100 ppm
N,N-dimethylacetamide	<5 ppm	1090 ppm
N,N-dimethylformamide	<20 ppm	880 ppm
1,4-dioxane	<20 ppm	380 ppm
2-ethoxyethanol	< 50 ppm	160 ppm
Ethylene glycol	< 50 ppm	620 ppm

Certificate of Analysis – Ceylon cinnamon

Formamide	< 50 ppm	220 ppm
Hexane	< 5 ppm	290 ppm
Methanol	< 100 ppm	3000 ppm
2-methoxyethanol	< 50 ppm	50 ppm
Methylbutylketone	< 5 ppm	50 ppm
Methylcyclohexane	< 5 ppm	1180 ppm
Methylene chloride	< 5 ppm	600 ppm
N-methyl-pyrrolidone	< 50 ppm	530 ppm
Nitromethane	< 5 ppm	50 ppm
Pyridine	< 50 ppm	200 ppm
Sulfolane	< 50 ppm	160 ppm
Tetrahydrofuran	< 5 ppm	720 ppm
Tetralin	< 50 ppm	100 ppm
Toluene	< 5 ppm	890 ppm
Trichloroethylene	< 5 ppm	80 ppm
Xylenes	< 5 ppm	2170 ppm
Methylisobutylketone	< 200 ppm	N/A

Class III – USP/NF 467 (modified)(Eurofins)

Residual solvent	Quantity	Allowable
Acetic acid	260 ppm	5000 ppm
Acetone	< 200 ppm	5000 ppm
Anisole	< 200 ppm	5000 ppm
1-butanol	< 200 ppm	5000 ppm
2-butanol	< 200 ppm	5000 ppm
Butyl acetate	< 200 ppm	5000 ppm
Tert-butylmethyl ether	< 200 ppm	5000 ppm
Dimethyl sulfoxide	< 200 ppm	5000 ppm
Ethanol	< 200 ppm	5000 ppm
Ethyl acetate	< 200 ppm	5000 ppm
Ethyl ether	< 200 ppm	5000 ppm
Ethyl formate	< 200 ppm	5000 ppm
Formic acid	< 200 ppm	5000 ppm
Heptane	< 200 ppm	5000 ppm
Isobutyl acetate	< 200 ppm	5000 ppm
Isopropyl acetate	< 200 ppm	5000 ppm
Methyl acetate	< 200 ppm	5000 ppm
3-methyl 1-butanol	< 200 ppm	5000 ppm
Methylethylketone	< 200 ppm	5000 ppm
2-methyl-1-propanol	< 200 ppm	5000 ppm
Pentane	< 200 ppm	5000 ppm
1-pentanol	< 200 ppm	5000 ppm
1-propanol	< 200 ppm	5000 ppm
2-propanol	< 200 ppm	5000 ppm
Propyl acetate	< 200 ppm	5000 ppm

Sum of Class 3 solvents < 5,000 ppm

Microbial Test Results

Listeria spp. – FDA BAM Chapter 10; *Salmonella* spp. – FDA BAM Chapter 5; *E. coli* – AOAC 991.14, ISO/IEC 17025:2017 A2LA 3329.04 (Eurofins)

Microbe	Result	Allowable
Listeria spp.	ND per 25 g	< 20 cfu/g
Salmonella spp.	ND per 25 g	< 20 cfu/g
Escherichia coli	< 10 cfu/g	< 20 cfu/g
